# Glucose Starvation Inhibits Ferroptosis by Activating the LKB1/AMPK Signaling Pathway and Promotes the High Speed Linear Motility of Dairy Goat Sperm

**DOI:** 10.3390/ani13091442

**Published:** 2023-04-23

**Authors:** Yu Li, Guangzhi Zhang, Fei Wen, Ming Xian, Songmao Guo, Xing Zhang, Xianzhou Feng, Zhangtao Hu, Jianhong Hu

**Affiliations:** Key Laboratory of Animal Genetics, Breeding and Reproduction of Shaanxi Province, College of Animal Science and Technology, Northwest A&F University, Yangling 712100, China; yuli0912@126.com (Y.L.); z13514592253@163.com (G.Z.); 13087561604@163.com (F.W.); xianmingtz@126.com (M.X.); 18854883308@163.com (S.G.); axingzzd66@126.com (X.Z.); fengxianzhou0930@163.com (X.F.); huzhangtao0819@163.com (Z.H.)

**Keywords:** dairy goat, sperm, glucose, mitochondrial oxidative phosphorylation, AMPK, ferroptosis

## Abstract

**Simple Summary:**

In mammals, sperm acquire fertilization ability after capacitation in vitro or when in the female reproductive tract. Adenosine triphosphate (ATP) is required for sperm capacitation through two main metabolic processes, oxidative phosphorylation (OXPHOS) and glycolysis. This study incubated dairy goat sperm with different concentrations of ROT, FCCP, TIG, and AMPK inhibitors. Sperm motility attributes, ATP content, pyruvate and lactate levels, mitochondrial permeability transition pore fluorescence intensity, MMP, protein synthesis, and ferroptosis were analyzed. The results showed that glucose starvation inhibited ferroptosis by activating the LKB1/AMPK signaling pathway and promoted the motility and linear motility of dairy goat sperm, thereby promoting fertilization.

**Abstract:**

In mammals, sperm acquire fertilization ability after capacitation in vitro or when in the female reproductive tract. The motility patterns of sperm undergo continuous changes from the moment of ejaculation until fertilization in the female reproductive tract. In vitro, hyperactivated motility can be induced through high glucose mediums, while in vivo, it is induced by oviduct fluids. Conversely, sperm maintain linear motility in seminal plasma or uterine fluids that contain low glucose levels. In dairy goat sperm, energy metabolism associated with capacitation depends on the energy sources in vitro, seminal plasma, or the female reproductive tract, especially the glucose levels. However, there is little experimental knowledge that glucose levels affect sperm energy metabolism in dairy goats. To clarify these hypotheses, we incubated dairy goat spermatozoa with different concentrations of rotenone-glucose (ROT), carbonyl cyanide 4-(trifluoromethoxy) phenylhydrazone (FCCP), and tigecycline (TIG) in vitro. Sperm motility attributes, ATP content, pyruvate and lactate levels, mitochondrial permeability transition pore fluorescence intensity, mitochondrial membrane potential (MMP), and protein synthesis were analyzed. Sperm motility patterns changed from circular to linear under low glucose conditions compared with those in high glucose conditions and showed a significant improvement in progressive motility and straight line speed, whereas lactate and pyruvate levels and MMP decreased remarkably. Incubation of spermatozoa with ROT, FCCP, and TIG inhibited sperm mitochondrial activity, protein synthesis, oxidative phosphorylation, and ATP levels, thereby reducing sperm motility, including the progressive motility, straight line speed, and total motility. Simultaneously, incubation of spermatozoa with Compound C under low glucose conditions significantly decreased the ATP levels and MMP, as well as liver kinase B1 and AMPK protein expression. Under low glucose conditions, sperm mainly produce ATP through mitochondrial OXPHOS to achieve high speed linear movement, inhibit ferroptosis through the LKB1/AMPK signaling pathway, and further maintain energy metabolism homeostasis.

## 1. Introduction

Sperm are highly specialized cells with unique compositional, morphological, and functional properties. After ejaculation, mammalian sperm acquire fertilization ability after capacitation in the female reproductive tract. Fertilization in mammals is a complex process influenced by multiple factors, especially because the site of ejaculation is at a distance from the site of fertilization [1]. Therefore, sperm migration, capacitation, hyperactivation, and acrosome reaction are all necessary for successful fertilization. In addition, the components in the female reproductive tract change during different reproductive stages. Under normal physiological conditions, the vagina mainly contains glucose, lactic acid, and glycerol, whereas the uterus and oviduct mainly contain glucose, pyruvic acid, and lactic acid [2]. When sperm enter the female reproductive tract, the components in the female reproductive tract are converted into fructose, citrate, glucose, lactic acid, and free amino acids [3]. These substrates of energy metabolism are necessary for spermatogenesis, maturation, and fertilization. As a substrate for sperm preservation in vitro, the glucose concentration should be in accordance with that in the female reproductive tract. Besides glucose, sperm also utilize other substrates such as fructose and sorbitol via glycolysis to maintain motility [4,5]. Sperm motility is driven by ATP from cytosolic glycolysis, mitochondrial OXPHOS, or both [6].

Sperm motility depends on the movement of sperm flagella and is driven by ATP production. Mitochondria are the most important organelles for ATP production in sperm. In human sperm, the ATP required for sperm motility is mainly generated from glycolysis. Although OXPHOS can produce ATP, it is not enough to sustain sperm movement during high motility [7]. Both glycolysis and OXPHOS are necessary to maintain vigorous motility. Incubation of mouse sperm in glucose-free media with a mitochondrial OXPHOS inhibitor (FCCP) and mitochondrial respiratory chain inhibitor (ROT) significantly reduced ATP levels and progressive motility [5,8,9]. Sperm motility parameters and the mitochondrial membrane potential (MMP) decrease significantly in sheep after treatment with FCCP and the glycolytic inhibitor 2-DOG, whereas sensitivity to lipid peroxidation (LPO) increases remarkably [10]. However, ATP production in horse sperm relies on mitochondrial OXPHOS in contrast to that in other mammals [11,12,13,14,15]. Therefore, sperm produce ATP for successful fertilization via different metabolic pathways in different energy conditions. However, the changes in motility patterns and selection of energy metabolic pathways for ATP production in dairy goat sperm in different energy conditions are unknown.

5′-AMP-activated protein kinase (AMPK) is a key regulator of energy homeostasis, which is achieved by stimulating ATP production or inhibiting ATP consumption to maintain energy homeostasis [16]. Glucose starvation is a classical activating condition of AMPK that maintains cell survival and redox homeostasis through multiple pathways [17]. In rat or mouse Sertoli cells, AMPK activation induces an increase in glucose uptake and lactate production, whereas the expressions of glucose transporter 1 (GLUT1) and monocarboxylic acid transporter 4 (MCT4) reduce significantly [18]. Platycodin D inhibits ferroptosis induced by high glucose in HK-2 cells, downregulates ACSL4 and TFR1 expressions, and upregulates FTH-1 and SLC7A11 expressions [19]. Furthermore, glycolysis and pentose phosphate pathways are also inhibited by glucose starvation, which induces oxidative stress that is characterized by an increased production of reactive oxygen species (ROS). This leads to interference with the antioxidant system, redox dysregulation, and cell death [20]. Hence, in this study, sperm motility patterns at different concentrations of glucose were measured, and sperm motility parameters were evaluated using a computer-assisted sperm analysis (CASA) system. We also determined the MMP, the opening fluorescence intensity of mitochondrial permeability transition pores (mPTP), and the protein expression after incubation with a mitochondrial respiratory chain inhibitor (rotenone), a mitochondrial OXPHOS inhibitor (FCCP), and a mitochondrial translation inhibitor (TIG) to confirm the metabolic pathways at different glucose concentrations. Lastly, we explored the relationship between ferroptosis-induced glucose starvation and oxidative damage.

## 2. Materials and Methods

### 2.1. Chemicals and Reagents

All chemicals and reagents were purchased from Sigma-Aldrich unless specified otherwise. All antibodies were used for incubation following the manufacturers’ instructions and were obtained from the following vendors: COX-1 (13393-1-AP, Proteintech, Wuhan, China), COXVB (11418-2-AP, Proteintech, Wuhan, China), ND3 (ab192306, Abcam, Cambridge, UK), NRF1 (12482-1-AP, Proteintech, Wuhan, China), SLC7A11 (26864-1-AP, Proteintech, Wuhan, China), GPX4 (67763-1-AP, Proteintech, Wuhan, China), DHODH (14877-1-AP, Proteintech, Wuhan, China), β-actin (20536-1-AP, Proteintech, Wuhan, China), horseradish peroxidase (HRP)-labeled goat anti-mouse immunoglobulin (Ig)G (CW0102S, CWBIO, Beijing, China), HRP-labeled goat anti-rabbit IgG (CW0103S, CWBIO, Beijing, China), and enhanced chemiluminescence (ECL) fluid (SuperSignal^TM^ West Femto Maximum Sensitivity Substrate, 34095, Thermo Fisher Scientific, Waltham, MA, USA).

### 2.2. Animals and Semen Collection

Semen was collected using an artificial vagina in Guan Zhong dairy goats (n = 5) from Shaanxi Aonick Dairy Goat Breeding Co., Ltd. (Weinan, China), that were raised under the same feeding condition as in Weinan, Shaanxi Province (34°76′ N, 109°17′ E). The semen of dairy goats was collected using the artificial vagina method and the collection frequency was three times a week on Monday, Wednesday, and Friday mornings. Semen samples with a deformity rate of less than 5%, normal morphology of sperm, motility greater than 0.8, and semen density exceeding 1.5 billion/mL were suitable for this study. Subsequently, the semen samples were pooled to eliminate individual variation and used for subsequent experiments.

### 2.3. Ethics Statement

All animal experiments were approved by the Animal Ethics Committee of Northwest A&F University. All experiments with dairy goats were implemented strictly according to the Guide for the Care and Use of Laboratory Animals.

### 2.4. Semen Processing

Dilute fresh sperm with a modified solution consisting of 172 mM glucose, 90 mM lactose, 58 mM sodium citrate, 1000 IU/mL penicillin G potassium, and 1 mg/mL streptomycin was passed through a 0.22 μm filter. The glucose concentration (172 mM) in the modified solution was determined to be 40%. A portion of lactose was used with glucose to prepare different doses of glucose bulking agents (0, 86, 172, 258, 344, and 430 mM, i.e., 0%, 20%, 40%, 60%, 80%, and 100%), as sperm do not use lactose as an energy substrate. Sperm were incubated with different concentrations of glucose medium at 37 °C for 1 h to evaluate whether dairy goat sperm were involved in the mitochondrial oxidative phosphorylation mode of energy supply. Moreover, sperm were incubated in a low glucose group containing 86 mM glucose and different concentrations of ROT, FCCP, and TIG at 37 °C for 5 h to evaluate the role of mitochondrial oxidative phosphorylation in the regulation of mitochondrial energy metabolism function in dairy goat spermatozoa. Finally, Compound C (AMPK inhibitor) was added to low and high glucose diluents and incubated in a water bath at 37 °C for 5 h to evaluate whether the low glucose dilutions activated AMPK to maintain energy homeostasis and inhibit sperm oxidative damage.

### 2.5. Sperm Motility

Sperm motility parameters were assessed using a CASA system (HVIEW-SSAV8.0, FuZhouHongShiYeSoftware Technology Co., Ltd., Fuzhou, China). Briefly, 10 μL of the samples was dropped onto a preheated glass slide and covered with a coverslip. Then, sperm motion was captured in at least five different regions using dynamic video acquisition of CASA on a heated platform at 37 °C. Each region was required to contain at least 200 sperm counts. Lastly, sperm motility parameters were assessed using the analysis software of CASA.

### 2.6. Biochemical Assays

All tests were conducted per the respective manufacturers’ instructions for each kit and were obtained from the following vendors: reactive oxygen species (ROS) assay kit (CA1410, Solarbio, Beijing, China), malondialdehyde (MDA) assay kit (BC0025, Solarbio, Beijing, China), pyruvate (PA) content assay kit (BC2205, Solarbio, Beijing, China), lactic acid (LA) assay kit (A019-2-1, Nanjing Jiancheng, Nanjing, China), ATP assay kit (S0026, Beyotime, Shanghai, China), MMP assay kit (M8650, Solarbio, Beijing, China), and bicinchoninic acid (BCA) protein assay kit (PA115, TAINGEN, Beijing, China). Finally, the related indexes were detected by a multifunctional enzyme label instrument (Synergy H1, American Berten, VT, USA).

### 2.7. Mitochondrial Permeability Transition Pore (mPTP) Fluorescence Intensity

Sperm samples were centrifuged at 1000× *g* at room temperature for 5 min and the supernatant was discarded. The sperm density was diluted to achieve a final concentration of 1 × 10^6^ sperm/mL by adding an appropriate volume of Calcein AM (1000×) staining solution and fluorescence quenching solution or ionomycin (200×) control, and incubated in the dark for 30 min. Then, the supernatant was removed by centrifugation at 1000× *g* at room temperature for 5 min and slowly resuspended twice at 37 °C. Subsequently, the samples were smeared and analyzed using flow cytometry (FACS Melody, BD Biosciences, Franklin Lakes, NJ, USA) within 1 h. The excitation wavelength was 494 nm and the emission wavelength was 517 nm.

### 2.8. Immunofluorescence Assays

Sperm samples were washed with phosphate-buffered saline (PBS), spread on a glass slide with poly-D-lysine, dried naturally, and fixed in 4% paraformaldehyde for 10 min. After drying, the sperm were penetrated with PBS containing 0.5% Triton X-100 for 30 min, washed three times for 5 min each, and blocked with 5% bovine serum albumin (BSA) in PBS-T for 30 min. Next, the slides were incubated with the primary antibody (1:100) overnight at 4 °C. Subsequently, the slides were incubated with the second antibody (1:200) at 37 °C for 1 h in the dark. The sperm nuclei were stained with DAPI at room temperature for 1 min in the dark and washed three times with PBS for 5 min each. Lastly, the samples were observed using a fluorescence microscope (LECIA-DM6 B, LECIA Co., Ltd., WETZLAR, Hessian, Germany).

### 2.9. Western Blotting

Protein concentrations were determined using a BCA protein assay kit (PA115, TAINGEN, Beijing, China). Proteins were separated using FuturePAGE™ 4–12% (ET15412Gel, ACE Biotechnology, Nanjing, China) sodium dodecyl sulfate–polyacrylamide gel electrophoresis, transferred to polyvinylidene fluoride membranes, and blocked with 5% BSA for 1 h at room temperature. The primary antibody was diluted and incubated with the membrane overnight at 4 °C. After incubation, the membrane was washed three times for 10 min each using Tris-buffered saline tween (TBST) and incubated with the second antibody at 37 °C for 1 h. After washing with TBST, detection was performed using enhanced chemiluminescence (ECL) (34095, Thermo Fisher Scientific, Waltham, MA, USA) using a Gel Doc XR System (BioRad, Hercules, CA, USA) according to the manufacturer’s specifications. Lastly, the intensity of the protein bands was analyzed using ImageJ software.

### 2.10. Statistical Analysis

Data are expressed as the mean ± standard error of the mean from at least 3 independent experiments. A statistical analysis among groups was performed using PRISM (version 6, GraphPad), followed by post hoc test using the Student’s *t*-test. The mean difference at *p* < 0.05 was considered statistically significant (* *p* < 0.05, ** *p* < 0.01, *** *p* < 0.001, and **** *p* < 0.0001. ns, with no apparent difference).

## 3. Results

### 3.1. Sperm Energy Metabolic Pathway and Sperm Motility Patterns Are Changed in Different Concentrations of Glucose

**Figure 1:**
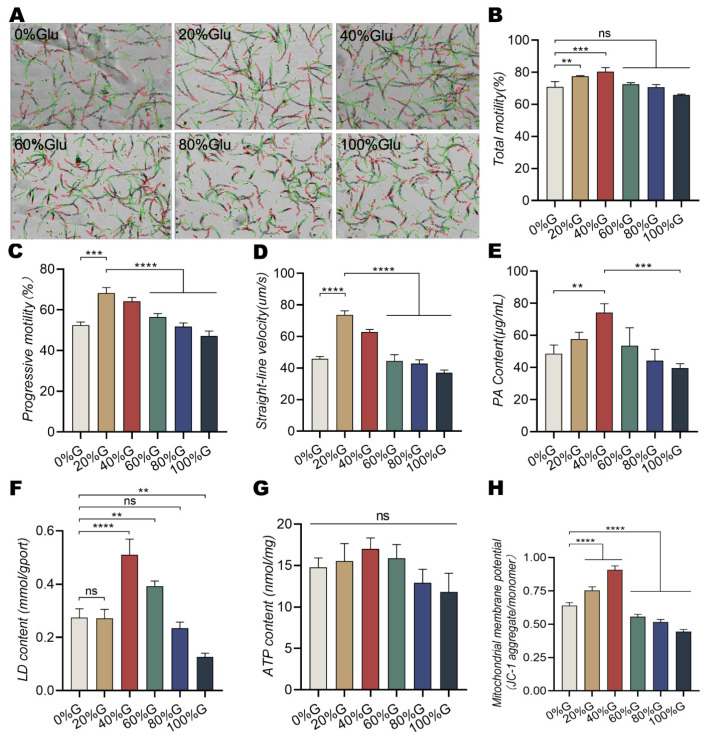
Effect of different concentrations of glucose on the motility and energy metabolism of dairy goat sperm. (**A**) Changes in sperm motility track using CASA. (**B**) Motility of sperm in dairy goats. (**C**,**D**) Progressive motility and straight line velocity of sperm of dairy goats. (**E**) PA content. (**F**) LD content. (**G**) ATP content in sperm. (**H**) Mitochondrial membrane potential. Values are presented as the mean ± standard error of the mean (SEM) of three replicates. Asterisks indicate statistical significance compared with the respective control groups. All results are expressed as the mean ± SEM, with asterisks indicating statistical significance for the respective control group. ** *p* < 0.01, *** *p* < 0.001, and **** *p* < 0.0001. ns, no significant difference.

The CASA system can detect the motility and movement trajectory of sperm. Sperm motility tracks changed into circle-like tracks in high glucose media (>60%), and the progressive motility and straight line velocity reduced significantly. However, the total motility and straight line velocities in the low glucose groups (20% and 40%) were significantly higher than those in the other groups (Figure 1A–D). To further determine the energy metabolic pathway, the levels of pyruvate, lactic acid, ATP, and MMP in the sperm of dairy goats were assessed at different glucose concentrations. The levels of pyruvate, lactic acid, and MMP were significantly higher in the 40% glucose group after incubation for 1 h compared with those in the control group, whereas the levels decreased remarkably in the high glucose group (Figure 1E–H, *p* < 0.001).

### 3.2. Addition of Rotenone to the Culture Reduces ATP Content and Straight Line Motility

**Figure 2:**
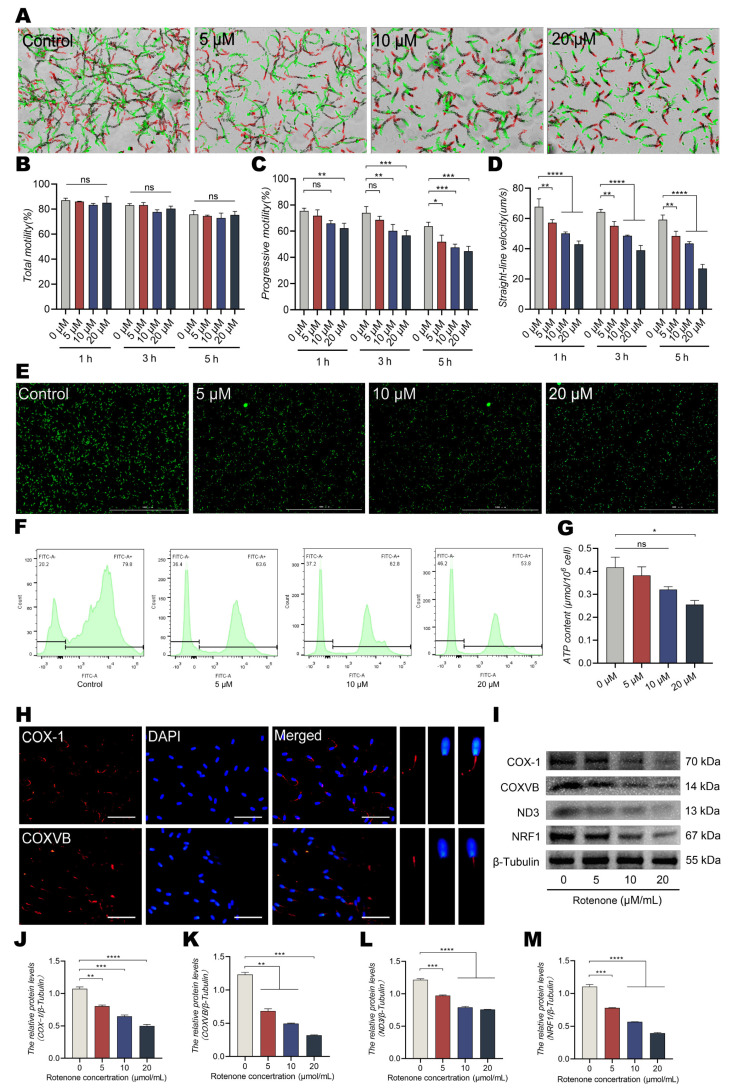
Incubation with rotenone reduces progressive motility and ATP levels. (**A**–**D**) Changes in sperm motility parameters evaluated using CASA: (**A**) sperm motility tracks, (**B**) motility of sperm of dairy goats. (**C**,**D**) Progressive motility and straight line velocity of sperm of dairy goats. (**E**–**H**) Effect of rotenone on sperm mitochondrial functions at the 5 h point: (**E**,**F**) opening fluorescence intensity of mitochondrial mPTP, (**G**) ATP content, and (**H**) immunolocalizations of COX-1 and COXVB in mitochondria. (**I**–**M**) Quantitative expression of COX-1, COXVB, ND3, and NRF1 using Western blotting after rotenone incubation for 5 h. All results are expressed as the mean ± standard error of the mean, with asterisks indicating statistical significance for the respective control group. * *p* < 0.05, ** *p* < 0.01, *** *p* < 0.001, and **** *p* < 0.0001. ns, no significant difference.

To further validate the role of OXPHOS in energy metabolism in dairy goat sperm, rotenone (an inhibitor of mitochondrial respiratory chain complex I) was incubated with sperm samples for 5 h in this study. The results from CASA showed significant reductions in forward sperm motility and straight line velocity, but no significant difference was observed in the total motility of sperm (Figure 2A–D). Moreover, there was a considerable decrease in the fluorescence intensity in a dose-dependent manner with respect to mPTP and ATP levels with the addition of rotenone (Figure 2E–G, *p* < 0.05). Therefore, the expression of the marker proteins of ETC and OXPHOS was determined using immunofluorescence staining and Western blotting. Immunofluorescence staining results showed that COX-1 and COXVB were mainly distributed in the middle segment of the sperm tails (Figure 2H) and that the expression of COX-1, COXVB, ND3, and NRF1 decreased significantly after treatment with rotenone (Figure 2I–M, *p* < 0.001). Taken together, it could be inferred that rotenone inhibits the regulation of mitochondrial OXPHOS in dairy goat sperm.

### 3.3. The OXPHOS Uncoupler FCCP Reduces Sperm Motility and ATP Levels

**Figure 3:**
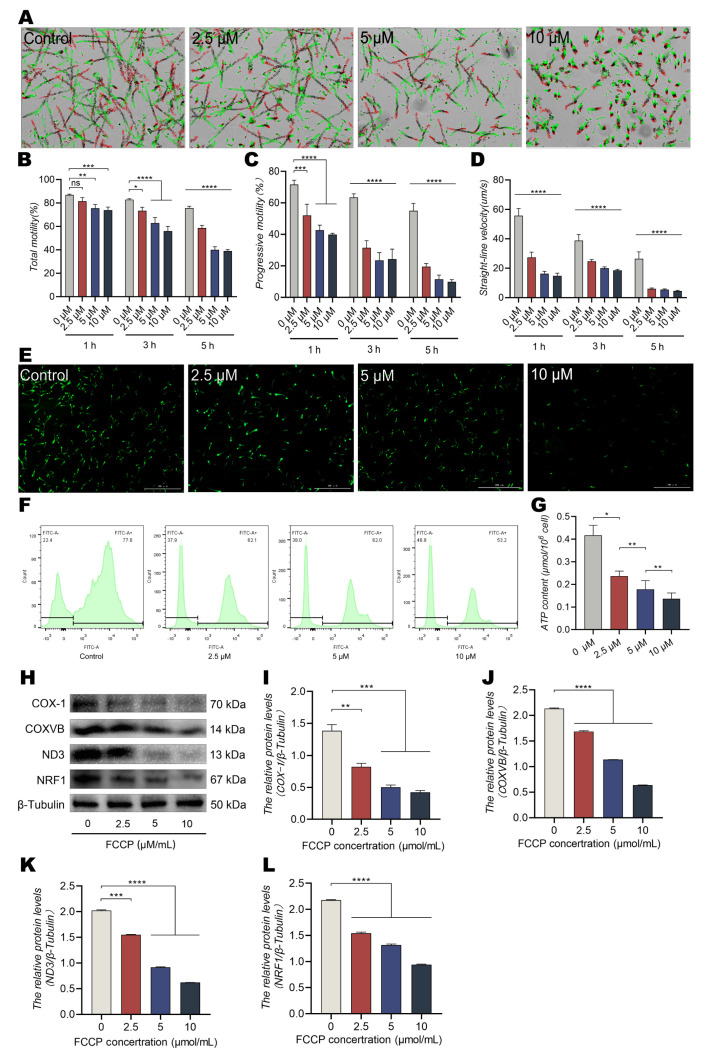
The oxidative phosphorylation uncoupler FCCP reduces sperm motility and ATP content. (**A**–**D**) Motility parameters of dairy goat sperm using CASA: (**A**) motility tracks were generated using CASA. (**B**) Motility of sperm of dairy goats. (**C**,**D**) Progressive motility and straight line velocity of sperm of dairy goats. (**E**–**G**) Effect of FCCP on sperm mitochondrial functions: (**E**,**F**) opening fluorescence intensity of mPTP in mitochondria and (**G**) ATP content. (**H**–**L**) Expressions of COX-1, COXVB, ND3, and NRF1 determined using Western blotting after treatment with FCCP for 5 h. All results are expressed as the mean ± standard error of the mean, with asterisks indicating statistical significance for the respective control group. * *p* < 0.05, ** *p* < 0.01, *** *p* < 0.001, and **** *p* < 0.0001. ns, no significant difference.

To further explore the effect of OXPHOS on energy metabolism in dairy goat sperm, the samples were treated with a specific inhibitor of mitochondrial OXPHOS (FCCP). The sperm motility, forward motility, and linear velocity significantly decreased in a dose-dependent manner after treatment with FCCP for 5 h (Figure 3A–D, *p* < 0.0001). The fluorescence intensity of mPTP and the level of ATP in mitochondria also decreased significantly (Figure 3E–G, *p* < 0.01). Furthermore, we observed that COX-1, COXVB, ND3, and NRF1 expressions in mitochondria decreased obviously (Figure 3H–L, *p* < 0.001).

### 3.4. The Mitochondrial Translational Inhibitor TIG Reduces MMP and ATP Levels in a Dose-Dependent Manner

**Figure 4:**
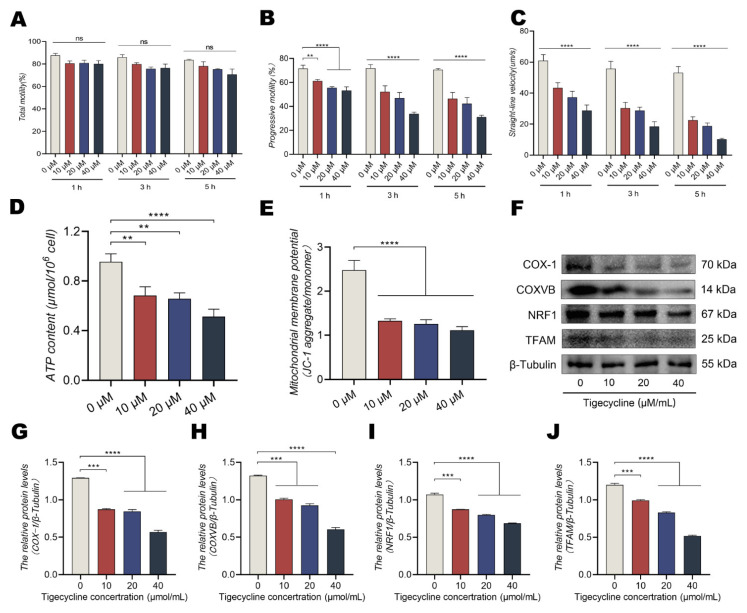
The mitochondrial translation inhibitor TIG decreases MMP and ATP levels in a dose-dependent manner. (**A**–**C**) Changes in sperm motility parameters using CASA: (**A**) motility of sperm of dairy goats. (**B**,**C**) Progressive motility and straight line velocity of sperm of dairy goats. (**D**) ATP levels in the mitochondria. (**E**) Mitochondrial membrane potential. (**F**–**J**) Protein levels of COX-1, COXVB, NRF1, and TFAM during 5 h of incubation with TIG determined using Western blotting. All results are expressed as the mean ± standard error of the mean, with asterisks indicating statistical significance for the respective control group. ** *p* < 0.01, *** *p* < 0.001, and **** *p* < 0.0001. ns, no significant difference.

To further confirm that the straight line motility pattern in dairy goat sperm relies on mitochondrial transcription and translation, the mitochondrial translation inhibitor TIG was incubated with sperm. There were no significant differences in sperm motility at the 5 h point, but the progressive motility and straight line velocity of sperm decreased significantly (Figure 4A–C, *p* < 0.0001). Moreover, ATP levels and MMP decreased noticeably after incubation with TIG (Figure 4D,E, *p* < 0.0001), indicating that the inhibition of mitochondrial translation reduces the progressive motility and straight line velocity of sperm. Additionally, COX-1, COXVB, NRF1, and TFAM expressions in mitochondria decreased significantly (Figure 4F–J, *p* < 0.0001). Therefore, energy metabolism and the linear motility pattern are closely related to transcription and translation in mitochondria.

### 3.5. Low Glucose Conditions Inhibit Ferroptosis-Induced Oxidative Damage by Regulating the Liver Kinase B1 (LKB1)/AMPK Signaling Pathway

**Figure 5:**
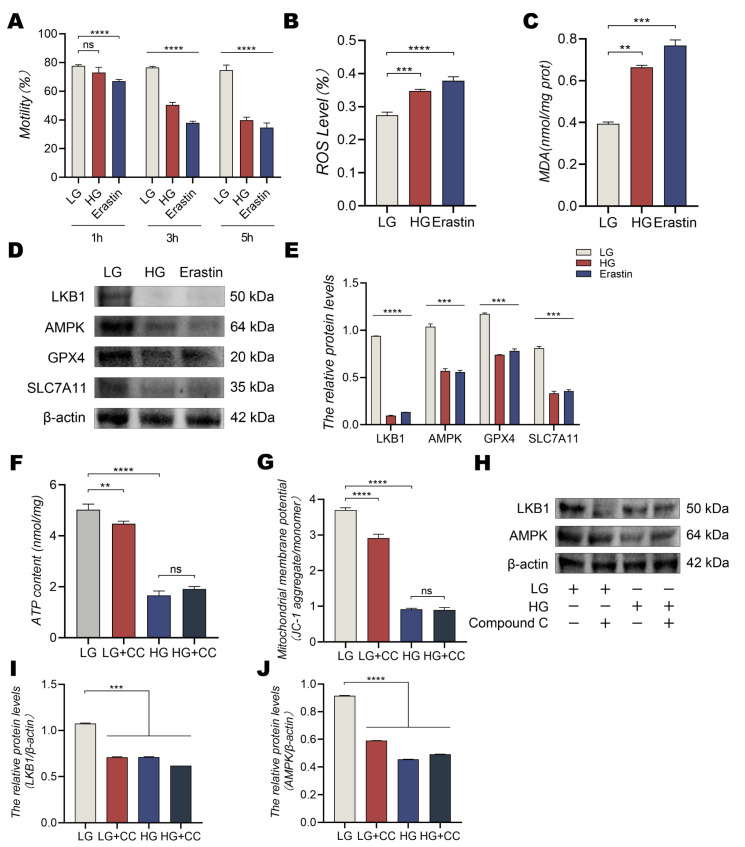
Low glucose conditions inhibit ferroptosis-induced oxidative damage by regulating the LKB1/AMPK signaling pathway. (**A**) Sperm motility. (**B**) ROS levels. (**C**) MDA levels. (**D**,**E**) Protein levels of LKB1, AMPK, GPX4, and SLC7A11 during 5 h of incubation in high glucose medium or with erastin determined using Western blotting. (**F**) ATP levels. (**G**) Mitochondrial membrane potential. (**H**–**J**) Expressions of LKB1 and AMPK after incubation with low or high concentrations of glucose and Compound C for 5 h. All results are expressed as the mean ± standard error of the mean, with asterisks indicating statistical significance for the respective control group. ** *p* < 0.01, *** *p* < 0.001, and **** *p* < 0.0001. ns, no significant difference.

High glucose media cause a reduction in cell viability as well as ROS accumulation, LPO, and outer mitochondrial membrane rupture, eventually inducing ferroptosis [21]. Therefore, low and high concentrations of glucose were used to determine whether low glucose conditions could inhibit ferroptosis and maintain the redox balance by activating AMPK in dairy goat sperm. Sperm motility in high glucose media did not change obviously compared with that in low glucose media at the 1 h point, as determined using CASA, but decreased significantly after high glucose or erastin incubation for 3 h or 5 h; moreover, ROS and MDA levels increased significantly (Figure 5A–C, *p* < 0.01). In addition, the intensities of LKB1, AMPK, glutathione peroxidase 4 (GPX4), and family 7 member 11 (SLC7A11) proteins decreased significantly in high glucose conditions or when treated with erastin (Figure 5D,E, *p* < 0.001). Therefore, high glucose levels could induce a reduction in sperm motility and lead to the accumulation of ROS and LPO in dairy goat sperm, which, in turn, caused ferroptosis. Glucose is the primary energy source for cell types, and AMP-activated protein kinase (AMPK) is an energy sensor. In low energy conditions, cells can be isomerically activated by adenosine monophosphate (AMP) and adenosine diphosphate [22]. Thus, an AMPK inhibitor (Compound C) was used to determine whether low glucose levels could inhibit ferroptosis in dairy goat sperm by regulating AMPK. A significant decrease was observed in ATP levels and MMP in the low glucose group (*p* < 0.01) but not in the high glucose group (Figure 5F,G). Furthermore, Western blotting revealed that the expression of LKB1 and AMPK proteins decreased noticeably in high glucose conditions or after treatment with Compound C (Figure 5H–J, *p* < 0.001).

## 4. Discussion

Mammalian sperm are produced through spermatogenesis and serve the purpose of fertilizing an oocyte. After ejaculation, sperm require energy to meet their demands. This energy can be obtained through the importation of exogenous substrates or the use of endogenous sources. Adenosine triphosphate (ATP), an important energy source that supports sperm capacitation and migration in the female reproductive tract, is generated from two main metabolic pathways, namely glycolysis and oxidative phosphorylation (OXPHOS). They occur in different compartments in sperm flagella, both of which are located in the principal piece [23]. The metabolic pathways of energy in sperm change in the presence of different metabolic substrates. In addition to these changes, the sperm motility pattern associated with capacitation also changes and is called hyperactivation [24]. The hyperactivated status is always characterized by a high curvilinear velocity and a high lateral amplitude [25]. However, the relationship between the energy metabolic pathway and motility patterns in dairy goat sperm remains unclear. In this study, we established a model by incubation of sperm with different concentrations of glucose and found that not only the progressive motility and straight line velocity of sperm but also ATP levels and MMP increased significantly in low glucose media. This finding indicated that sperm regulate the energy metabolism pathway based on changes in the metabolic substrates and that low glucose levels activate OXPHOS in the mitochondria to maintain the normal functions of sperm. In terms of the changes in oxygen and substrate levels in the female reproductive tract, sperm adapt to the fluctuations in exogenous substrate levels via different energy metabolism pathways [26]. Qiu et al. found that among the substrates tested, glucose and pyruvate were better than lactate at maintaining goat sperm motility. Pyruvate enters goat spermatozoa through monocarboxylate transporters and is oxidized by the tricarboxylic acid cycle and electron transfer to sustain sperm motility [27]. Zhu et al. reported that GSK3α/β was expressed in the head acrosome and the mid, main end of the tail of goat sperm and that it regulated goat sperm motility and the acrosome response by mediating energy pathways in glycolysis and oxidative phosphorylation [28]. Thus, these different energy metabolic pathways are interconnected in sperm, and different concentrations of glucose can selectively activate glycolysis as well as OXPHOS in the mitochondria. Moreover, pyruvate and lactate are involved in the glycolysis pathway. In this study, the progressive motility of sperm was found to be regulated by OXPHOS in the mitochondria, which is similar to the physiological state of sperm in the female reproductive tract after ejaculation. The semen, uterus, and oviduct contain high levels of pyruvate, lactate, and amino acids, and sperm can utilize these metabolites through OXPHOS for energy metabolism, further enhancing their progressive motility and speed of access to reach the site of fertilization. Zhu et al. demonstrated that ATP generated from OXPHOS in the mitochondria in low glucose conditions induced progressive motility in boar sperm. On the other hand, incubation with CRP, a mitochondrial translation inhibitor, inhibited mitochondrial translation in sperm and decreased their progressive motility but not total motility, suggesting that motility patterns in sperm depend on the substrates of energy metabolism [29]. Moreover, high glucose concentrations led to a transient inefficiency in the late stages of both glycolysis and NADH accumulation and decreased the conversion flux from fructose-1,6-bisphosphate to pyruvate and the ATP content [30].

Mitochondria are called the motive source of cells and play a key role in energy metabolism through the tricarboxylic acid (TCA) cycle, β-oxidation, and OXPHOS [31]. During glycolysis, a glucose molecule produces two ATP molecules, whereas in the TCA cycle or OXPHOS pathway, a glucose molecule produces 32 ATP molecules. OXPHOS is composed of four respiratory complexes (complexes I to IV) and ATP synthase (complex V) and plays a key role in MMP across the mitochondrial inner membrane [32]. Rotenone is a strong inhibitor of complex I of the mitochondrial respiratory chain. Heo et al. demonstrated that rotenone induces mitochondrial dysfunction by inhibiting SIRT1 during oocyte maturation in pigs in vitro, thereby reducing mitochondrial activity and ATP generation and increasing ROS production [33]. There was no significant difference in sperm motility after incubation with rotenone, but there was a significant decrease in mitochondrial activity and ATP levels [29]. Besides, FCCP is a classic uncoupler of mitochondrial OXPHOS. Davila et al. showed that sperm motility, speed, and the number of live sperm decreased significantly in horses after incubation for 3 h with FCCP, but the MMP and ATP levels were unaffected [15]. Blanco-Prieto et al. have reported that FCCP significantly increases oxygen consumption and decreases the total motility of boar sperm [34]. In this study, dairy goat sperm were incubated with rotenone and FCCP separately. After treatment with rotenone, their total motility did not change much, but the progressive motility and straight linear speed reduced significantly. The addition of FCCP decreased the overall survival and mPTP permeability in a dose-dependent manner. Both these treatments significantly reduced the MMP and ATP content. Furthermore, the protein expression related to the mitochondrial respiratory chain determined using Western blotting showed that incubation with rotenone and FCCP resulted in the disruption of the ETC in the mitochondria and inactivation of ATP synthase [35]. On the other hand, the relationship between mitochondrial function and ATP generation from OXPHOS is especially close, and mitochondrial activity is influenced by proteins from the mitochondrial respiratory chain. Zhu et al. found that in a low glucose environment, 13 genes involved in mitochondrial transcription and translation were remarkably upregulated, which, in turn, improved mitochondrial OXPHOS and progressive motility in boar sperm [29]. Consistent with the above findings, tigecycline (a mitochondrial translation inhibitor) inhibited the expression of proteins in the mitochondrial respiratory chain in our study, decreased the MMP and ATP content, and reduced the progressive motility of dairy goat sperm. Glucose and fructose are essential components as a semen preservation diluent, but excessive glucose induces cellular damage [36]. High glucose concentrations induce cell apoptosis, ferroptosis, necrosis, or other types of cell death [37,38]. Ferroptosis is a new form of oxidative cell death that is induced by small molecules and especially by the imbalance of lipid ROS in cells [8]. Ferroptosis is also induced by other factors, including the inhibition of glutathione synthetase or GPX4, leading to the accumulation of LPO products and ROS and eventually resulting in cell death [39]. In our study, incubation with either high glucose levels or erastin for 3 h significantly reduced sperm motility, increased ROS and MDA levels, and decreased the expressions of LKB1/AMPK, SLC7A11, and GPX4. However, in low glucose media, there was no significant difference in sperm motility after incubation for 3 h, indicating that the normal functions of sperm could be maintained by accelerating ATP production through the AMPK pathway [40]. Glucose starvation is a typical activator of AMPK and an important regulator in energy homeostasis. The LKB1/AMPK signaling pathway is critical in maintaining cell survival under low glucose conditions [20]. Metformin (an activator of AMPK) was found to reduce the levels of LPO-stimulated steroid, restore spermatogenesis, and increase sperm motility in the testes of diabetic or obese rats [41]. During cryopreservation of chicken sperm, the addition of an AMPK activator, metformin or AICAR, improved AMPK phosphorylation after thawing, whereas LPO and ROS production was reduced [42]. Additionally, the activation of AMPK in goat sperm improved sperm motility, plasma membrane integrity, and the acrosome reaction; maintained normal levels of MMP, lactate acid, and ATP; and enhanced the activity of AMPK, PK, and lactate dehydrogenase. However, the addition of Compound C inhibited the AMPK pathway and induced the opposite effects [43]. In the present study, the addition of Compound C to the low glucose media led to reductions in ATP levels and MMP as well as a decrease in LKB1/AMPK expression compared with that in the high glucose group. These results suggested that the LKB1/AMPK signaling pathway can be activated to maintain energy homeostasis in sperm during energy stress.

## 5. Conclusions

Energy metabolism in dairy goat sperm is realized through the mitochondrial OXPHOS pathway under low glucose conditions, and the high speed linear motility and straight line velocity improved significantly. Moreover, in this study, the results of incubation with rotenone and FCCP confirmed that the progressive motility of sperm mainly relies on OXPHOS in the mitochondria and that ATP generation from OXPHOS is closely related to mitochondrial functions. Low glucose conditions promote transcription and translation in the mitochondria and activate mitochondrial OXPHOS to supply energy to sperm. Moreover, the LKB1/AMPK pathway is activated to maintain energy homeostasis and inhibit ferroptosis-induced oxidative damage. Therefore, the high speed linear motion induced by low sugar is a new factor to improve insemination in artificial insemination of livestock and human fertility treatments. As a convenient and inexpensive method, sperm dilution with a low-sugar insemination solution improves the rapid linear movement of sperm within the female reproductive tract, thereby facilitating fertilization.

## Data Availability

The authors confirm that the original contributions presented in this study are reflected in this manuscript.
